# A multidimensional study for design of phytochemical profiling, antioxidant potential, and enzyme inhibition effects of ışgın (*Rheum telianum*) as an edible plant

**DOI:** 10.1016/j.fochx.2024.102125

**Published:** 2025-01-05

**Authors:** Ahmet Zafer Tel, Kubra Aslan, Mustafa Abdullah Yılmaz, İlhami Gulcin

**Affiliations:** a Department of Agricultural Biotechnology, Faculty of Agriculture, Igdir University, 76000 Igdir, Türkiye; b Department of Chemistry, Faculty of Science, Ataturk University, 25240 Erzurum, Türkiye; c Faculty of Pharmacy, Department of Pharmaceutical Chemistry, Dicle University, 21280 Diyarbakır, Türkiye

**Keywords:** *Rheum telianum*, Antioxidant activity, Acetylcholinesterase;α-glycosidase, Rhubarb, Phenolic compound

## Abstract

The present study reveals *in vitro* antioxidant properties and the phytochemical content of a novel *Rheum* species (*Rheum telianum*), which grows in Southeastern Anatolia. To perform the analysis, dried leaves and seeds of the plants were ground and extracted with ethanol to obtain plant secondary metabolites and antioxidant activity. Then, dried extracts were subjected to *in vitro* DPPH scavenging and Cupric reducing (CUPRAC), Fe^3+^, and Ferric reducing antioxidant power (FRAP). In addition to the antioxidant capacity assays, quantitative phenolic, flavonoid, and secondary metabolite were determined through spectrophotometric and LC-MS/MS chromatographic methods. IC_50_ values showed that both leaves and the seeds of the *R. telianum* have high inhibitory properties over DPPH radicals with 20.79 and 5.67 μg/mL, respectively. The samples' dominant secondary metabolites were evaluated through the LC-MS/MS analysis results. The inhibition effects of both leaves and the seeds of the *R. telianum* extracts on acetylcholinesterase and butyrylcholinesterase, α-glycosidase and human carbonic anhydrases II isoenzyme enzymes, which associated with some global diseases including Alzheimer's disease, type-2 diabetes mellitus and glaucoma were determined. In conclusion, the extracts' contents and functional relationship and the plants' possible usage in the food, medicine, and cosmetic industries was revealed.

## Highlights

• Total phenolic and flavonoids in ışgın (*Rheum telianum*) were determined.
• Antioxidant activity of ışgın (*Rheum telianum*) was evaluated using several bioassay.
• Anticholinergic effect of ışgın (*Rheum telianum*) was determined and compared to Tacrine.
• Antidiabetic effect of ışgın (*Rheum telianum*) was realized and compared to Acarbose.
• Antiglaucoma effect of ışgın (*Rheum telianum*) was defined and compared to Acetazolamide.

## 1 Introduction

Food and medicinal plants, which have vital importance for human diet and health. They are indispensable food sources and an integral part of traditional medicine systems worldwide. These plants have held an important place in human health and food culture since ancient times. Although more than 35,000 plant species have been identified for this purpose, the repertoire and reservoir of biomolecules useful to humanity have not yet been fully filled and have not reached to sufficient biological potentials (Baretto & Sahebkar, 2021). Thousands of plants are added to the plant spectrum used for this purpose every year. One of the plants recently added to this plant collection, which has both medicinal and food value, is ışgın (*Rheum telianum* Ilçim).

Işgın (*Rheum* spp.), rhubarb in folk linguistics, is a genus that species-rich type of the Polygonaceae family. This genus is mostly native to Asian countries like China, Nepal, India, Korea, Pakistan, Bhutan, Iran, Turkey, Tibet and Russia. Currently, there are sixty-one accepted different species, among 33 of those species have been reported to have biological importance. Among them *R. australe*, *R. ribes*, *R. palmatum*, and *R. webbianum* have the highest distribution in the world. These species have important usage in traditional medicine as they have been reported to be positive effective in liver, kidney, gastrointestinal, and reproductive system-related diseases. Also, due to their proven biological activity, they have a great potential to be used as sources in herbal medicine (Baretto & Sahebkar, 2021.; Bennur et al., 2024; Ilçim & Karahan, 2020; Liudvytska et al., 2023; Takeoka et al., 2013). Işgın (*R. telianum*) is a novel rhubarb species that is s described as a new plant species from Kayatepe village (SouthEastern Anatolia, Turkey). This plant is morphologically related to the West-Central Asian plants *R. rhizostachyum* and *R. ribes* (Ilçim & Karahan, 2020). Here, it was aimed to determine the antioxidant properties and phytochemical content of this novel *R. telianum* to evaluate its biological activity relativeness to other important *Rheum* sp. and provide an insight into possible future biological activity with this preliminary study.

Food and medicinal plants are the main sources of natural antioxidants that help to improve the properties of cosmetics, foods, and medicines, as well as for nutritional purposes and disease prevention, and are of great interest to consumers today (Adugna et al., 2024; Çakmakçı et al., 2015). These medicinal and food plants are also sources of phytochemicals with strong biological activities and many beneficial effects due to their strong antioxidant effects (Kızıltaş et al., 2021). Reliable research has shown that supplementary antioxidants reduce the excessive generation of free radicals and reactive oxygen species (ROS), keep them under control and play a critical role in healthy living (Çetinkaya et al., 2012; Zhang et al., 2024). ROS are formed during oxidative stress, and endogen antioxidants in the body and exogenous antioxidants taken with the diet control their formation and production (Topal & Gulcin, 2022). The imbalance between antioxidant levels and ROS in metabolism enhances the oxidative stress risk and, in this case, different diseases begin to occur (Durmaz et al., 2024). So, preventing of ROS formation may be a vital importance in reducing some chronic disease including, cancer, diabetes and inflammation (Bingol et al., 2021). There is a great interest and need for natural additives and antioxidants to protect food products against oxidative deterioration and harmful ROS effects on metabolism, instead of commonly and generally used synthetic antioxidants such as buthylated hydroxytoluene (BHT), buthylated hydroxyanisole (BHA), and *tert*-butylhydroquinone (TBHQ) (Elmastas et al., 2006). After the scientific link between BHA and BHT and liver damageand cancer development were discovered, the interest in synthetic antioxidants gradually decreased and serious restrictions were imposed on their use (Balaydin et al., 2010).

According to the International Diabetes Federation reports diabetes mellitus is estimated to cause more than 7 million deaths worldwide each year (Gulçin et al., 2018). Type-2 diabetes mellitus (T2DM) is the most common diabetic type and is characterized by insulin resistance and requires ongoing medical attention (Gulcin et al., 2022). T2DM, defined as persistent hyperglycemia, has been associated with insulin resistance, obesity, and a systemic inflammation called meta inflammation, which damages β-cells in the pancreas (Bursal et al., 2019). On the other hand, α-glycosidase is a digestive enzyme that hydrolyzes complex carbohydrates into monomeric glucose units. It plays a pivotal role in the regulation of postprandial hyperglycemia (Petrova et al., 2023). α-Glycosidase is very important for the digestion of dietary polysaccharides. The inhibition of α-glycosidase had great importance in the diabetes treatment for the well-being of diabetic patients (Eruygur et al., 2019). α-Glycosidase activity remains a promising strategy for regulating blood glucose levels (Zhang et al., 2024). Also, α-glycosidase inhibitors (AGIs) significantly inhibit the absorption of monosaccharides in the small intestine by inhibiting the breakdown of polysaccharides to monosaccharide units. In this case, a decrease in postprandial blood plasma glucose levels occurs. Therefore, naturally derived AGIs can be used to treat global and widespread diseases, such as diabetes and obesity (Bursal et al., 2019). Today, there are many studies in the field of AGIs synthesis (Taslimi et al., 2017; Taslimi & Gulçin, 2017). Among the different classes of compounds from natural sources, many natural products with α-glycosidase inhibition effects have been found. Especially nowadays, the interest in plant-derived AGIs is increasing (Lam et al., 2024). After the years, the α-glycosidase inhibitory effects of natural product have been obtained in numerous studies (Durmaz et al., 2022; Durmaz, Gulçin, et al., 2023; Durmaz, Kiziltas, et al., 2023).

Alzheimer's disease (AD) is a neurodegenerative disease that is usually seen in the elderly and manifests itself with abnormal behaviors, forgetfulness, cognitive dysfunctions, deficiencies in language and mental retardation (Günsel et al., 2024; Topal et al., 2017). AD is pathologically characterized by nerve fiber tangles in nerve cells, loss of nerve cells, senile plaques between nerve cells, and synaptic changes (Zhai et al., 2024). However, although there are many studies on the pathogenesis of AD, it has not yet been fully elucidated. The important role of acetylcholinesterase (AChE) in the nervous system is considered as the main aberrant pathway. The main AChE function is to hydrolyze acetylcholine (ACh) to acetate (CH_3_COO^–^) and choline (Ch) molecules (Genc Bilgicli et al., 2020; Özbey et al., 2016). When AChE content is high and activity is strong, ACh content decreases rapidly. In this case, neuronal damage occurs and AD formation is caused. Therefore, oral drugs that can inhibit the existing AChE content in the brain can easily alleviate the symptoms of AD patients (Karlsson et al., 2012). So, the use of AChE inhibitors (AChEIs) for presentation of the cholinergic breakdown of ACh is a prospective and strong strategy to treat AD (Cetin Cakmak & Gülçin, 2019). Another important reason of AD development is the increase in the amount and of amyloid-β (Aβ) accumulation in the brain. There is an effective Aβ cleaning system in the human body. In this system, proteolytic enzymes in the brain parenchyma break down Aβ. For this reason, Aβ is transported to the cerebrospinal fluid and plasma for a further breakdown process. In this way, the Aβ level in the body becomes relatively stable. When Aβ is examined, it is seen that there are three different forms. These are Aβ_1-40_, Aβ_1-42_ and Aβ_1-43_. Among these, the Aβ_1-42_ oligomer is the most dangerous form, has a powerful toxic effect and damage on nerve cells and triggers increased aggregation of Aβ fibers. Accumulation of Aβ, a neurotoxic substance, causes not only neuronal degeneration but also many pathological events, including destruction of the blood-brain barrier (BBB), activation of astrocytes and microglia, and changes in microcirculation. These pathological conditions are the main causes of neuronal degeneration in AD patients (Jellinger, 2022; Lau et al., 2021; Zhai et al., 2024).

Carbonic anhydrases (CAs) are zinc ions (Zn^2+^) containing metalloenzymes and catalyze the reversible hydration of carbon dioxide (CO_2_) and water to protons (H^+^) and bicarbonate (HCO_3_^−^), which occurs by enzymatic mechanism (Göksu et al., 2014; Nar et al., 2013). They exist in prokaryotes and eukaryotes and encoded by eight different and unrelated gene families (Koçyiğit et al., 2018; Özbey et al., 2016). CAs play a various of biochemical processes including lipogenesis, gluconeogenesis and ureagenesis (Genc Bilgicli et al., 2020). They also continue a fluid balance in body, particularly in the eyes. Glaucoma disease-related enhanced intraocular pressure (IOP) can be treated with CA inhibitors (Goçer et al., 2015; Turkan et al., 2019).

In this present study, we investigated the phytochemical profiling, antioxidant potential, and enzyme inhibition effects of ışgın (*R*.*telianum*) as a novel edible plant species collected from the South-Eastern region of Anatolia. For this purpose, the following studies was performed: (i) total quantity of phenolics and flavonoids, which determined by Folin-Ciocalteu and Al(NO_3_)_3__3_ assays; (ii) the quantity of major phenolic and flavonoid compounds of ışgın were evaluated using LC-MS/MS; (iii) the antioxidant capabilities of ışgın were measured using by ferric ions (Fe^3+^) reducing, cupric ions (Cu^2+^) reducing (CUPRAC), ferric-reducing antioxidant power (FRAP), and DPPH radical scavenging effects, and (iv) the inhibitory effect of ışgın on some metabolic enzymes including AChE, BChE, hCA II and α-glycosidase, which associated with diabetes mellitus, AD, and glaucoma, were determined and compared to clinical inhibitors.

## 2 Materials and methods

### 2.1 Chemicals

1,1-Diphenyl-2-picryl-hydrazyl (DPPH) radicals, neocuproine (2,9-dimethyl-1,10-phenanthroline), ascorbic acid, BHT, BHA, α-tocopherol, Trolox, Folin Ciocalteu reagent and Ellman's reagent (5,5-dithio-bis-(2-nitrobenzoic acid) were purchased from Sigma-Aldrich GmbH, Steinheim, Germany. Standard phenolic compounds for LC-MS/MS were purchased from Sigma.

### 2.2 Preparation of the plant samples

*R.telianum* leaves and the seeds were collected from Kayatepe (Rezip) village of Adıyaman province in Türkiye, rocky serpentine soils, 37°87’ N, 38°27’ E, 1390 m TURKEY (Ilçim & Karahan, 2020). Water and ethanol extracts of the samples were prepared by weighing 10 g of dried leaves and the seeds after grinding pieces up to 0.5–10 mm. Then, 50 mL of ethanol was added to the milled plant material in a beaker and the samples were mixed for 5 hours. Solid particles were decanted from both clean cheesecloths, and Whatman paper No.1, and centrifugated at 3000 rpm for 10 minutes until transparent extracts were obtained. The extracts were evaporated through dryness (Karagecili, İzol, et al., 2023; Karagecili, Yılmaz, et al., 2023). The overall extraction yields were calculated from the remaining extracts. 100 mg of dry extracts were separated for LC-MS/MS analysis and ethanol solution of extracts was prepared in known concentrations for further antioxidant capacity testing.

### 2.3 Determination of total phenolics in extracts

The total phenolic contents of the plant extracts were evaluated by the Folin–Ciocalteu method applied by Karagecili et al. splitting the volumes of each component by half (Karagecili, İzol, et al., 2023; Karagecili, Yılmaz, et al., 2023). The results were expressed as μg gallic acid equivalents (GAE) /mg of the extracts with concentrations ranging between 0 and 200 μg/mL (y=0.007993x – 0.1509, r^2^= 0.9824).

### 2.4 Determination of total flavonoid and total phenol of the extracts

Total phenolics contents were determined by analyzing through the colorimetric method (Karagecili, İzol, et al., 2023; Karagecili, Yılmaz, et al., 2023). Different concentrations in 0.5 mL of the samples in ethanol were mixed with 1.5 mL of 95% methanol. Then, 0.5 mL of 1.0 M potassium acetate, 2.3 mL of distilled water, and 1.5 mL of 10% aluminum nitrate were added to the reaction mixture, and the samples were incubated at 25°C for 40 min after vigorously shaking. Absorbances at 415 nm of each reaction were recorded and the results were expressed as μg quercetin equivalents (QE) /mg of the extracts. A standard curve of quercetin was obtained within concentrations in range of 1–500 μg/mL (y=0.004614x+0.01149, r^2^=0.9909).

### 2.5 Determination of phytochemicals by LC-MS/MS analysis

Screening of ethanol extract of the samples was performed against 53 standard phytochemicals whose chromatographic conditions were validated and optimized for A Shimadzu-Nexera model ultrahigh performance liquid chromatography (UHPLC) coupled with a tandem mass spectrometer (Shimadzu LC/MS-8040) in the previously reported study (Yilmaz, 2020). LC-MS/MS method was applied as previously reported (Karagecili, İzol, et al., 2023; Karagecili, Yılmaz, et al., 2023) and adopted for dried samples of ethanol extract of *R*. *telianum* leaves (EERL), and the *R*. *telianum* seeds (EERS).

### 2.6 DPPH radical scavenging activity

The DPPH radical scavenging activity determination assay was performed as previously applied (Blois, 1958) An ethanol solution of 100 μM DPPH was prepared and incubated in the dark by mixing overnight for pre-radicalization. Then, 0.5 mL of DPPH and 0.5 mL of the samples in ethanol (15-45 μg/mL) were mixed and incubated at 30 °C for 30 minutes. The absorbances of each sample were recorded at 517 nm. Ascorbic acid, BHA, BHT, α-Tocopherol, and Trolox were used as positive controls in this assay. Each sample was performed in triplicate.

### 2.7 Fe^3+^reducing ability capacity

Fe^3+^-reducing assay was employed to determine the ability of the samples to reduce metal ions (Gülçin et al., 2004). Three different concentrations of the samples (0.75 mL in distilled water) were mixed with 1.25 mL of 0.20 M phosphate buffer solution (pH 6.6) and 1% (w/w) potassium ferrocyanide. Then, the mixture was acidified with 1. 25 mL of 10% trichloroacetic acid (w/w), and incubated at 50°C for 30 min. Lastly, 0.25 mL of 0.1% FeCl_3_ was added to form the blue complex and the absorbances of each sample were recorded at 700 nm via Shimadzu UV-1800 UV spectrophotometer (Karagecili, İzol, et al., 2023; Karagecili, Yılmaz, et al., 2023).

### 2.8 Cu^2+^reducing ability capacity

CUPRAC assay was performed by combining an equal volume of 10 mM of CuCl_2_, 7.5 mM neocuproine, and 1.0 M ammonium acetate buffer and the samples were added by adjusting the total volume to 2 mL with deionized water (Gülçin, 2008). The mixture was incubated for 30 min at 25 °C and was measured spectrophotometrically using the blue-colored final reaction mixture at 450 nm.

### 2.9 FRAP reducing ability capacity

FRAP reagent containing acidic 10 mM: 20 mM FeCl_3_: 0.3 M sodium acetate buffer (pH 3.6) in a ratio of 1:1:10 was prepared before use. 0.5 mL of the samples in buffer mixed with an equal volume of 20 mM FeCl_3_ and FRAP reagent resulting in 5 mL final reaction volume. The absorbance of each reaction was measured at wavelength 593 nm after 30 min incubation at 37 °C (Bursal & Gülçin, 2011). BHT, ascorbic acid, BHA, Trolox and α-Tocopherol were used as positive controls in FRAP assay. Each sample was performed in triplicate.

### 2.10 Acetylcholinesterase and butyrylcholinesterase inhibition assay

Extract solutions at varying concentrations to complete inhibition were added to a mixture containing an equal 10 mM 5,5’-dithio-bis (2-nitro-benzoic) acid (DTNB) and 10 mM substrate in 1 mL of the reaction solution (Gocer et al., 2016; Köse et al., 2015).The substrate was acetylcholine iodide for AChE and butyrylcholine iodide for BChE. Right after enzyme addition (10 μL), the absorbance at 412 nm of the mixture was measured for five minutes at minute interval. Control reactions and blank reactions were set up without inhibitors and enzymes. Positive control for AChE and BChE inhibition test was donepezil (Scozzafava et al., 2015; Yamali et al., 2018).

### 2.11 α-Glycosidase enzyme inhibition assay

The inhibitory effects of the extracts on α-glycosidase were determined based on the method as given in detail (Eertmans et al., 2014). Different amounts of EERL and EERS were transferred to phosphate buffer (0.1 M, pH 6.9) for this purpose. Then, 20 μL of α-glycosidase enzyme solution was added to the same buffer and mixed with 50 μL of p-nitrophenyl-D-glycopyranoside (p-NPG). The absorbances of each sample were measured at 405 nm for 3 minutes towards a blank sample consisting of phosphate buffer instead of the sample. The control reaction was the sample without extracts.

### 2.12 Human carbonic anhydrases II isoenzyme (hCA II) inhibition assay

Carbonic anhydrase II isoenzyme (CA II) was purified by Sepharose 4B-L-Tyrosine-Sulfanilamide affinity column chromatography and its purity is determined by SDS-PAGE as previously applied (Verpoorte et al., 1967) as described previously (Güney et al., 2014). The esterase activity of the CA II isoenzyme yields a yellow-colored aromatic p-nitrophenolate compound pH around 7.5. By taking advantage of this transformation, the esterase activity of CA II isoenzyme can be determined spectrophotometrically. Various concentrations of the plant samples in 0.05 M pH=7.4 Tris-SO_4_, 0.07 mM of p-nitrophenylacetate (in 1:25 acetone: water), and 20 μL of the enzyme were gently vortexed, and as soon the as the addition of the enzyme, the absorbance change at 348 nm was monitored by measuring minute intervals for three minutes. Control reactions and blank reactions were set up without inhibitors and enzymes. These steps were repeated until more than half of enzyme activity was inhibited.

### 2.13 IC_50_ determination

Extract concentrations causing 50% enzyme inhibition (IC_50_) were calculated from activity (%) vs. extract concentration graphs. For this purpose, enzyme inhibition was studied at various extract concentrations. The values obtained were plotted as % activity against extract concentrations. IC_50_ values were calculated from the non-linear regression model of normalized inhibition responses to increased inhibitor concentration (Gulcin & Alwasel, 2022).

### 2.14 Statistical analysis

All experiments performed in the study were repeated three times for each sample. Results were recorded as mean ± SD (n = 3). One-way ANOVA was used followed by Tukey's post hoc test. Finally, p < 0.05 was considered statistically significant.

## 3 Results and discussion

### 3.1 Phenolic contents of ışgın (*Rheum telianum*) extract

Phytochemicals found in plants, especially phenolic substances, are vital and basic components of human nutrition. It is of great interest and importance due to the wide range of biological activities resulting from the antioxidant effects of the molecules in these dietary components EERL and EERS contain some phenolic compounds such as hesperidin, rutin and quercitrin, which have positive impacts on human health. Such phenolic compounds also inhibit lipid oxidation and increase the shelf life and chemical stability of the food product (Kızıltaş et al., 2021; Topal & Gulcin, 2022). Gallic acid, catechin, gentisic acid, rutin, quercitrin isoquercitrin, hesperidin, nicotiflorin and, acacetin are among the most abundant components in EERL and EERS. These components strengthen the antioxidant capacity and immune system in metabolism, and accordingly, increase the metabolic and physiological abilities in human body (Ak & Gülçin, 2008). Phenolic compounds, which are found in almost all fruits and vegetables and are also called secondary metabolites, are the most important natural components. Therefore, these components, which are commonly found in plants, are important exogenous molecules in the human diet. Phenolics in plants may vary depending on the distribution and altitude of the plant, maturity and variety of the plant, season, geographical origin and post-harvest storage conditions (Akyüz, 2022).

The total quantity of phenolic and flavonoid compounds in ethanol extracts of ışgınleaves (EERL) and seeds (EERS) were evaluated. Also, plant secondary metabolites in EERL and EERS were determined through LC-MS/MS against 53 phytochemical standards (thirty-three medicinal and 10 aromatic compounds) that were most commonly reported with their biological activity (Yilmaz, 2020). Since the significant biological effects such as antimicrobial, antioxidant, and anti-inflammatory properties of natural compounds were associated with phenolic contents. Their analysis is inevitable for food, medicine, and cosmetic industries when determining the value of the herbal extract (Karagecili, İzol, et al., 2023). According to the results obtained from the quantitative assays, the total phenolic content of EERL and EERS was calculated as 583 and 753 μg GAE /mg. In a recent study, 11.364 μg gallic acid equivalents phenolic compound was found in ethanolic extract of the aerial parts of *Salvia macrochlamys* (Kızıltaş et al., 2024). On the other hand, 96.00 and 6250 mg/mg GAE phenolic compounds was detected in methanol extract of *Thymus canoviridis* and *Thymus pubescens* (Güven et al., 2024). Even though the flavonoids have same application field as phenolic compounds such as cosmetic and food industry, the most pronounced applications of these compounds, are in the field of medicine. Therefore, the determination of the total flavonoid content will significantly express the medicinal value of the plant extract (Yaşar et al., 2021). The total flavonoids were also found as 298 and 238 QE μg/mg. Plant-to-extract ratios were defined by simple calculation of the extract production yield of dried plants. Based on this assumption, the dry extracts ratio of the leaves and the seeds was calculated as 10:5.9 and 10:3.9, respectively. Kızıltaş et al. (2024) detected 1.598 μg quercetin equivalent flavonoids in ethanolic extract of the aerial parts of *S*. *macrochlamys* (Kızıltaş et al., 2024). Also, 111.80 and 86.23 μg/mg QE flavonoids was found in methanol extract of *T*. *canoviridis* and *T*. *pubescens*, respectively (Güven et al., 2024; Bulut et al., 2023).

**Fig. 1:**
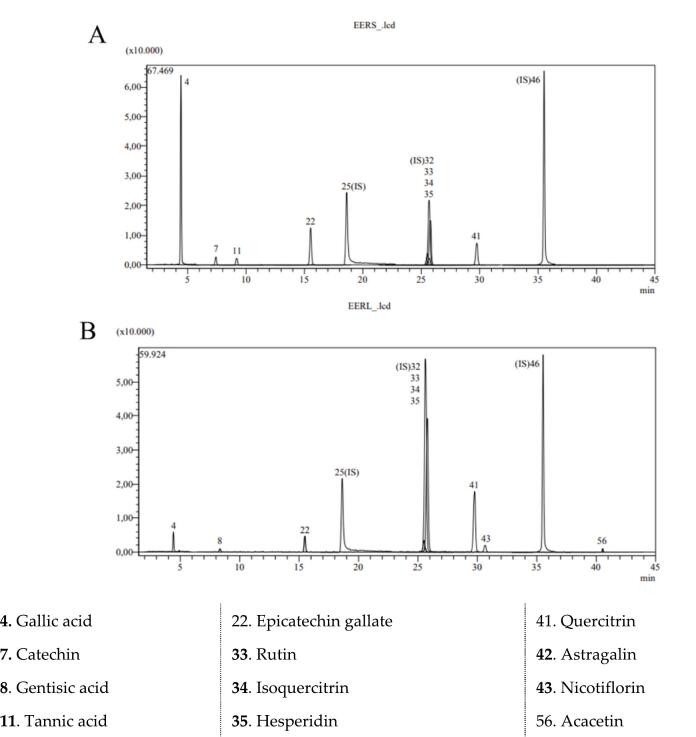
A. Chromatogram of used standard phenolics in *R*. *telianum* analyzed by LC-MS/MS analysis. **A**: Leaves of *R*. *telianum* (EERL) and **B**: the seeds of *R*. *telianum* (EERS).

LC-MS/MS analysis of the EERL and EERS revealed a total of ten different compounds in the leaves and the seeds with high amounts. In the EERL, gallic acid gentisic acid, epicatechin gallate, rutin, isoquercitrin, hesperidin quercitrin, nicotiflorin, and acacetin; in the seeds gallic acid, catechin, gentisic acid, epicatechin gallate, rutin, isoquercitrin, hesperidin, and quercitrin were detected. Even though the quantities differed, a total of eight compounds were in common in both plant extracts. Quantitative and chromatographic representations of the results are given in Fig. 1. The major phenolic components recorded in EERL were the rutin (0.868 mg/g), catechin (0.552 mg/g) and hesperidin (0.519 mg/g). On the other hand, the most abundant phenolic components were calculated as rutin (2.149 mg/g), hesperidin (1.357 mg/g) and quercetin (0.984 mg/g) in the EERS (Table 1). In another work, hispidulin (5469.62 mg/kg), hederagenin (3993.77 mg/kg), rosmarinic acid (3919.65 mg/kg) was calculated as the most abundant phenolic compounds in ethanolic extract of the aerial parts of *S*. *macrochlamys* (Kızıltaş et al., 2024). Furthermore, the phenolic compound composition of methanol extract of *T*. *canoviridis* was determined in descending order of concentration, with rosmarinic acid (18285.55 μg/g extract), quinic acid (1902.67 μg/g extract), and chlorogenic acid (220.24 μg/g extract) identified as the primary components. Similarly, rosmarinic acid (2910.59 μg/g extract), keracyanin chloride (1629.75 μg/g extract), chlorogenic acid (841.91 μg/g extract) in methanol extract of *T*. *pubescens* (Güven et al., 2024).

**Table 1:** LC-MS/MS quantitative and chromatographic results of ethanolic extract of ışgın (*R*. *telianum*) leaves (EERL) and the seeds (EERS).

Compounds	**Analytes**	**Phenolics (mg/g extracts)**	
		**EERS**	**EERL**
**4**	Gallic acid	1.000	0.090
**7**	Catechin	0.552	N.D.
**8**	Gentisic acid	0.020	0.027
**22**	Epicatechin gallate	1.120	0.109
**33**	Rutin	0.868	2.149
**34**	Isoquercitrin	0.117	0.026
**35**	Hesperidin	0.519	1.357
**41**	Quercitrin	0.430	0.984
**43**	Nicotiflorin	N.D.	0.034
**56**	Acacetin	N.D.	0.030

IS: internal standard, N.A.: not applicable, N.D.: not detected.

### 3.2 Reducing abilities results

In the present study, which examining an endemic plant, some common and effective bioanalytical methods such as phenolic contents determination, DPPH· scavenging and reducing abilities were used for measurement the antioxidant capacity of EERL and EERS. The reductive potentials of EERL and EERS were elucidated using different and distinct reductive systems, including Cu^2+^, Fe^3+^, and Fe^3+^-TPTZ reducing capabilities. Also, for the revealing of radical scavenging effect of the EERL and EERS were determined using only DPPH radical scavenging method.

The antioxidant ability of the ışgın plant was investigated through four different methods with two different mechanisms since combining different methods would be a more realistic approach regarding different antioxidant properties (Gulcin, 2020). The DPPH· scavenging effects of ethanol extract of leave and seeds of ışgın was determined, and the half maximal inhibition concentration (IC_50_) value was calculated (Table 2). EERL and EERS exhibited dose-dependent radical scavenging effects. Here, radical removing activity was determined by employing the DPPH radical scavenging method, and IC_50_ was calculated by simply portioning with the control reaction that does not involve the samples (Gulcin & Alwasel, 2023). Results showed that both extracts scavenged the DPPH radicals up to 90%, and even the seed extracts reached up to 98% (IC_50_: 5.67 μg/mL), which is greater activity than those ascorbic acid (IC_50_: 13.92 μg/mL), BHA (IC_50_: 16.00 μg/mL), BHT (IC_50_: 12.99 μg/mL), and Trolox (IC_50_: 9.63 μg/mL), were inhibited maximum with 94%. IC_50_ values of each sample in the DPPH assay were calculated using GraphPad Prism 8.4.0 using a non-linear regression model for normalized parameters. Mean absorbance values obtained from the metal-reducing tests were also tested by row statistics. IC_50_ results were obtained for samples and represented in Table 2. In a recent study, it was determined that ethanolic extract of the aerial parts of *S*. *macrochlamys* exhibited IC_50_ value of 86.63 (r^2^: 0.9945) against DPPH radicals (Kızıltaş et al., 2024).

**Table 2:** Half maximal radical scavenging concentration (IC_50_, μg/mL) of ethanol extract of seed and leave of ışgın (*R*. *telianum*) leaves (EERL) seeds (EERS) and standard molecules for DPPH radical scavenging activity.

Antioxidants	DPPH radical scavenging activity	
	IC_50_	r^2^
BHA	16.00	0.9948
BHT	12.99	0.9883
Trolox	9.63	0.9988
Ascorbic acid	13.92	0.9989
EERL	20.79	0.9884
EERS	5.67	0.9969

**Fig. 2:**
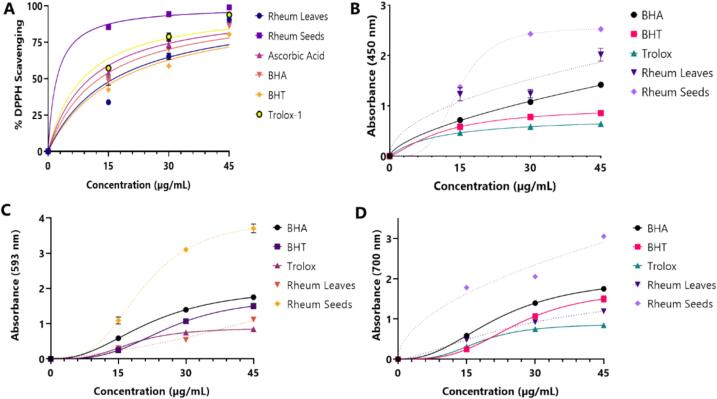
Graphical representation of antioxidant capacity results of leaves and seeds of ışgın (*R*. *telianum*) extracts. **A**; DPPH radical removing assay **B**; CUPRAC assay **C**; FRAP assay **D**; Fe^3+^-reducing assay.

The reducing capacity of EERL and EERS was evaluated using the FRAP test (The Fe^3+^-TPTZ ability). Due to the colored combination of Fe^3+^-TPTZ, this test reflects spectrophotometrically a maximum absorbance at 593 nm (Köse et al., 2015). Depending on the reducing effects of EERL and EERS, the yellow test solution color changed to different green or blue colors in this method. In Fe^3+^-TPTZ reducing results, the order was evaluated from the highest to the lowest, such as EERS, BHT, Trolox, BHA, and EERL. The second reduction method we used for evaluation of the antioxidant effect of our endemic plant's extracts is the ferric ions (Fe^3+^) reduction method. This method is widely used especially to directly estimation of the antioxidant ability of plant extracts. In the experimental studies, the addition of EERL or EERS to solutions containing Fe^3+^ ions results in the creation of blue colored Fe_4_[Fe(CN^-^)6]_3_ that can absorb light at 700 nm (Öztaşkin et al., 2015). The presence of plant extracts causes the formation of the green colored chromophore complex, which shows the reducing ability of the plant extracts on different shades from yellow to green. Secondly, the order was EERS, BHA, BHT, EERL, and Trolox from the highest to lowest in Fe^3+^-reducing assay. A graphical representation of the antioxidant capacity results is shown in Fig. 2. In the third reduction power test we used, EERL and EERS and the standards demonstrated effective Cu^2+^ reductive capacities. The Cu^2+^ reduce capacity of various EERL and EERS concentrations were shown to be positively correlated. EERL and EERS's ability to reduce cupric ions was found as concentrations-dependently (15–45 μg/mL). In addition to the easy applicability of this method, it is also very cheap and can be done in a short time.

Reducing capacities of ethanol extract of leave and seeds of ışgın were determined through three different methods and quite similar results were obtained from each of the assays. In first reducing effect method, the ferric ion (Fe^3+^) reductive abilities of EERL and EERS were determined according to the method, which described previously (Kızıltaş et al., 2021). As a result of the addition of Fe^3+^ to the solvent medium in which both extracts are present, Fe_4_[Fe(CN^–^)_6_]_3_ complex is formed and the complex exhibits maximum absorbance at 700 nm. As given in Table 3 and Fig. 2D, the EERL and EERS demonstrated a potent Fe^3+^ reducing effects. And also, the Fe^3+^ reducing effects of a concentration 30 μg/mL of EERL, EERS and standards decreased in the following order: EERS (3.07±0.03, r^2^: 0.9725) > BHA (1.75±0.04, r^2^: 0.9983) > BHT (150±0.08, r^2^: 0.9972) > EERL (1.19±0.02, r^2^: 0.9979) > Trolox (0.84±0.01, r^2^: 0.9990). All analyses were done in triplicate. In the spectrophotometric measurements, if the absorbance was more than 1.5, the samples were diluted in order to obtain accurate measurements in accordance with the Lambert-Beer law. Also, the results were multiplied by the dilution factor. In a recent study, it was determined that ethanolic extract of aerial parts of *S*. *macrochlamys* demonstrated the absorbance of 0.88 ± 0.02 (r^2^: 0.9645) in Fe^3+^ reducing ability at the similar concentration (Kızıltaş et al., 2024).

**Table 3:** The reducing abilities of ethanol extract of leave and seeds of ışgın (*R*. *telianum*) and standard molecules at the concentration of 45 μg/mL.

Antioxidants	Fe^3+^ reducing		Cu^2+^ reducing		FRAP reducing	
	λ_700_	r^2^	λ_700_	r^2^	λ_700_	r^2^
BHA	1.75 ± 0.04	0.9983	1.41 ± 0.07	0.9948	2.33 ± 0.01	0.9986
BHT	1.50 ± 0.08	0.9972	0.86 ± 0.02	0.9989	2.87 ± 0.06	0.9938
Trolox	0.84 ± 0.01	0.9990	0.64 ± 0.01	0.9988	2.48 ± 0.12	0.9558
EERL	1.19 ± 0.02	0.9979	2.02 ± 0.13	0.9272	1.12 ± 0.07	0.9789
EERS	3.07 ± 0.03	0.9725	2.53 ± 0.01	0.9997	3.70 ± 0.12	0.9981

The Cu^2+^-reducing capacity of the EERS was evaluated as being the highest and EERL, BHA, BHT, and Trolox were followed that result in descending order. The cupric ion (Cu^2+^) reducing effects of EERL and EERS are summarized in Table 3 and Fig. 2B. A strong correlation was found between the Cu^2+^ reducing ability and different EERL and EERS concentrations. In addition to this correlation, at 45 μg/mL, effective reducing ability was demonstrated by the EERL and EERS. Also, Cu^2+^ reducing ability of EERL, EERS and the standard molecules were calculated to be EERS (2.53±0.01, r^2^: 0.9997) > EERL (2.02±0.13±0.07, r^2^: 0.9272) > BHA (1.41±0.07, r^2^: 0.9948) >BHT (0.86±0.02, r^2^: 0.9989) > Trolox (0.64±0.01, r^2^: 0.9988). It was observed that EERS reduced more effectively cupric ions than all positive controls used in the study. As explained in the previous method, samples with absorbance values higher than 1.5 in spectrophotometric measurements were diluted to obtain accurate measurements in accordance with the Lambert-Beer law. In addition, the results were multiplied by the dilution factor. In this test, the higher absorbance values reflect the greater the reducing ability of the test samples (Gülçin et al., 2019). Recently, it was found that ethanolic extract of aerial parts of *S*. *macrochlamys* demonstrated the absorbance of 0.840±0.020 (r^2^: 0.9772) for Cu^2+^ reducing ability at the similar concentration (Kızıltaş et al., 2024). The absorbance value at a concentration of 30 μg/mL was found as 0.373 and 0.566 for methanol extracts of *T*. *pubescens* and *T*. *canoviridis*, respectivelly (Güven et al., 2024).

In Fe^3+^-TPTZ reducing results, the order was evaluated from the highest to the lowest, such as EERS, BHT, Trolox, BHA, and EERL. Lastly, the order was EERS, BHA, BHT, EERL, and Trolox from the highest to lowest in Fe^3+^-reducing assay. A graphical representation of the ferric ions reducing antioxidant capacity results is shown in Fig. 2C and Table 3, which provide an overview of the determination into EERL and EERS's Fe^3+^-TPTZ reducing abilities in addition to its Fe^3+^ and Cu^2+^-reducing effects. At the used concentrations, EERL and EERS had effective high absorption level. The following order was found, EERL and EERS, and reduced Fe^3+^-TPTZ at a 45 μg/mL: EERS (3.70±0.12, r^2^: 0.9981) > BHT (2.87±0.06, r^2^: 0.9938) > Trolox (2.48±0.12, r^2^: 0.9558) > BHA (2.33±0.01, r^2^: 0.9986) > EERL (1.12±0.07, r^2^: 0.9789). In another study, it was determined that ethanolic extract of aerial parts of *S*. *macrochlamys* demonstrated the absorbance of 0.840±0.020 (r^2^: 0.9817) in Fe^3+^-TPTZ reducing ability at the similar concentration (Kızıltaş et al., 2024). The absorbance at 30 mg/mL was found as 0.373 and 0.566 for methanol extracts of *T*. *pubescens* and *T*. *canoviridis*, respectivelly (Güven et al., 2024).

### 3.3 Radical scavenging results

Here, radical scavenging activity was determined by employing the DPPH radicals, and the inhibition percentage was obtained by simply portioning with the control reaction that does not involve the samples. Results showed that both samples scavenged the DPPH radical up to 90%, and even the seed extracts reached up to 98%, which is greater activity than those ascorbic acid, BHA, BHT, Trolox were inhibited maximum with 94%. Reducing capacities were determined through three different methods and quite similar results were obtained from each of the assays. The Cu^2+^-reducing capacity of the EERS was evaluated as being the highest and EERL, BHA, BHT, and Trolox were followed that result in descending order.

The DPPH^•^ removing effects of EERL and EERS was measured, and their IC_50_ values was determined (Fig. 2A, Table 3). EERL and EERS exhibited dose-dependently radical removing ability. EERL and EERS revealed effective anti-radical abilities (IC_50_: 20.79 μg/mL, r^2^: 0.9884 and IC_50_: 5.67 μg/mL, r^2^: 0.9969). The results reveal that EERS had stronger DPPH radical removing than BHA (IC_50_: 16.00 μg/mL, r^2^: 0.9948), ascorbic acid (IC_50_: 13.92 μg/mL, r^2^: 0.9989), BHT (IC_50_: 12.99 μg/mL; r^2^: 0.9948), and trolox (IC_50_: 9.63 μg/mL, r^2^: 0.9988). On the other hand, EERL had a lover antioxidant ability when compared to all positive controls. At all EERL and EERS concentrations, the DPPH^•^ level decreased significantly (*p*<0.01).

### 3.4 Enzyme inhibition results

The CA isoenzymes inhibition has been intensively investigated recently and their inhibitor potential as drugs has been demonstrated for some diseases (Gulçin & Taslimi, 2018). In this context, one of the most studied isoenzymes, CA II, has been associated to glaucoma, edema, epilepsy, and altitude sickness, and has led researchers to conduct intensive studies (Hashmi et al., 2021). Important therapeutic goals have been achieved in the treatment of diseases in this context Suppression of CA II activity inhibits aqueous humor secretion by reducing the formation of HCO_3_ˉ ions, ultimately resulting in lower interocular pressure (IOP) (Biçer et al., 2019). Glaucoma, which occurs mostly in association with high IOP, is a multifactorial optic disease characterized by optic nerve degeneration and can lead to blindness. Although clinically used CA II inhibitors like acetazolamide, dorzolamide and brinzolamide are highly effective in lowering IOP level after topical treatment, intensive studies are being conducted to design and synthesize safer inhibitors with fewer side effects (Akbaba et al., 2014; Çelik Onar et al., 2023).

**Fig. 3:**
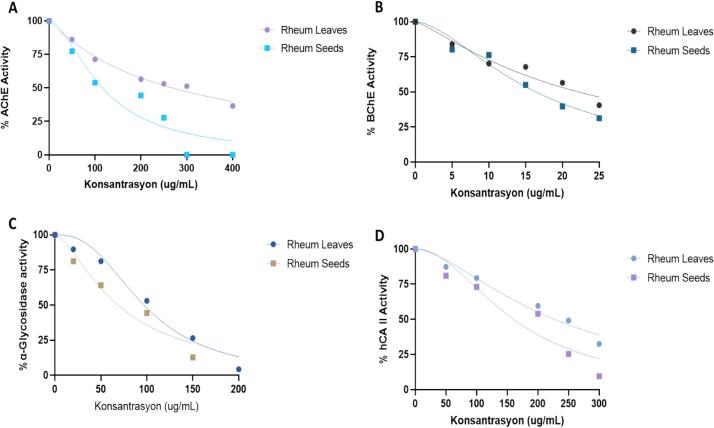
Graphical representation of enzyme inhibitory properties of ışgın (*R*. *telianum*) leaves (EERL) and the seeds (EERS) extracts against **A**: Acetylcholinesterase, **B**: Butyrylcholinesterase, **C**: α-Glycosidase, **D**: human carbonic anhydrase II.

Enzyme inhibition results ethanolic extracts ışgın leaves (EERL) and the seeds (EERS) showed that both EERL and EERS have inhibition effect on the AChE, BChE, α-glycosidase, and hCA II isoform. It is known that the hCA II isozyme, which is the most abundant free protein in human metabolism after hemoglobin, is associated with some metabolic diseases, especially glaucoma, renal tubular acidosis and osteoporosis. CA II inhibitory effects of EERL, EERS and acetazolamide were determined and the results are summarized in Fig. 3D and Table 4, IC_50_ values were recorded as 227.30 μg/mL (r^2^: 0.9712) for EERL and 160.50 μg/mL (r^2^: 0.9158) for EERS against dominant and cytosolic CA II isozyme. This value was observed as 9.96 μg/mL (r^2^: 0.9921) for clinical standard of acetazolamide, that used as a positive control for the CAs inhibitory experiment (Gümüş et al., 2022). The ethanolic extract of the aerial parts of *S*. *macrochlamys* was found to have an IC_50_ value of 0.530 μg/mL (r^2^: 0.9655) on α-glycosidase, as reported by Kızıltaş et al. (2024).

**Table 4:** Half maximal inhibition concentration (IC_50_, μg/mL) of ışgın (*R*. *telianum*) leaves (EERL) and the seeds (EERS) extracts against human carbonic anhydrase isoenzyme II (hCA II) acetylcholinesterase (AChE), butyrylcholinesterase (BChE), and α-glycosidase enzymes.

Antioxidants	hCA II		AChE		BChE		α-Glycosidase	
	IC_50_	r^2^	IC_50_	r^2^	IC_50_	r^2^	IC_50_	r^2^
EERL	227.30	0.9712	268.70	0.9874	22.20	0.9567	97.03	0.9725
EERS	160.50	0.9158	116.60	0.9168	16.41	0.9669	68.64	0.9524
Acetazolamide^1^	9.96	0.9921	–		–	–	–	–
Tacrine^2^	–	–	8.82	0.9765	15.51	0.9743	–	–
Acarbose^3^	–	–			–	–	9.43	0.9874

^1^Acetazolamide was used standard for hCA II.

^2^Tacrine was used standard for AChE and BChE.

^3^Acarbose was used standard for α-glycosidase, which obtained from literature (Tao et al., 2013).

An important population-based study has shown that elderly patients with diabetes have a higher risk to develop Alzheimer's disease (AD) and that there is a link between diabetes and age-related neurodegeneration (Wang et al., 2012). Such studies indicate that severe impairments in glucose metabolism due to diabetes play a key role in the onset and progression of AD. It has also been reported that in cases of hyperglycemia, reactive oxygen species (ROS) are formed in the form of stimulation of the polyol metabolic pathway, which leads to advanced glycation end products and induces oxidative stress (Gülçin, 2006). In this case, it is very important that the natural components taken with the diet have both antioxidant effects and anti-glycemic properties. In the advanced stages of AD, AChE and BChE enzymes are responsible and linked enzymes (Turan et al., 2016). Among the elderly, AD is the most common neurodegenerative disorder and the most important cause of dementia. The decrease in brain AChE level is the most important biochemical parameter in AD (Yiğit et al., 2022). In this context, cholinergic enzyme inhibitors are used on a large scale in the treatment of AD. However, some negative side effects of these drugs have been identified up to now. Based on these findings, new, and powerful inhibitors and their sources are of great importance (Bora et al., 2022). It has been reported that plants with medicinal or food value are particularly rich in cholinesterase inhibitors. Phenolic compounds found in these plants, which also have antioxidant effects, have been reported to suppress cholinergic enzymes (Taslimi et al., 2020). In the current research, the cholinesterase inhibitory abilities of EERL and EERS were determined. In particular, EERS was observed to inhibit both AChE and BChE to a greater extent.

AChE inhibitory effects of the EERL and EERS was determined and compared to tacrine, as positive control for cholinesterase inhibition. Table 4 and Fig. 3A exhibits the IC_50_s of EERL and EERS. The results showed middle inhibition level for EERL (IC_50_: 268.70 μg/mL, r^2^: 0.9874) and EERS (IC_50_: 116.60 μg/mL, r^2^: 0.9168). Although EERL showed lower AChE inhibitory effect when compared to EERS, however, it was observed that the AChE inhibitory effect of both extracts was significantly lower than that of Tacrine (IC_50_: 8.82 μg/mL, r^2^: 0.9765), which was utilized as a positive control for the cholinergic enzyme inhibition experiments. IC_50_ values were also recorded for comparison the inhibitory abilities of all applications against BChE enzymes (Table 4, Table 3B). As given in Table 4, IC_50_ values of EERL and EERS for BChE inhibition were 22.20 μg/mL (r^2^: 0.9657) and 4.78 μg/mL (r^2^: 0.9669), respectively, whereas, as shown in the same Table 4, this inhibition value for Tacrine towards BChE was 15.51 mg/mL, (r^2^: 0.9743). It was reported that ethanolic extract of the aerial parts of *S*. *macrochlamys* exhibited IC_50_ value 1.622 (r^2^: 0.9818) against AChE (Kızıltaş et al., 2024).

Diabetes is a chronic and highly prevalent metabolic disease characterized by hyperglycemia, which occurs as a result of inadequate insulin production by the pancreas or inadequate utilization of insulin by the metabolism, or a combination of both (Karagecili, Yılmaz, et al., 2023; Kızıltaş et al., 2025). Relative suppression or inhibition of α-glycosidase activity leads to a delay in the digestion and absorption of sugars in the small intestine. For this purpose, α-glycosidase inhibitors such as miglitol and acarbose are clinically used. Clinical and experimental studies have proven that these molecules provide higher insulin sensitivity and lower postprandial hyperglycemia. The action mechanism of such inhibitors is to block the α-glycosidase enzyme in the small intestine that managing for the digestion of complex carbohydrate polymers. In this process, polymeric carbohydrate hydrolysis and glucose absorption are reduced, and postprandial and postnatal blood glucose levels are reduced (Artunc et al., 2020; Nilofar et al., 2024).

These enzyme inhibitors help keep postprandial glucose levels low by preventing the digestion of carbohydrate polymers such as starch and glycogen. In this way, postprandial hyperglycemia is controlled. EERL and EERS displayed IC_50_ values of 97.03 μg/mL (r^2^: 0.9725) and 68.64 μg/mL (r^2^: 0.9524) on α-glycosidase (Table 4 and Fig. 3C). The results show that EERL and EERS has lower inhibitory properties than acarbose (IC_50_: 9.43 nM, r^2^: 0.9874) as α-efficient and clinical glycosidase acarbose (Tao et al., 2013). The ethanolic extract of the aerial parts of *S*. *macrochlamys* was found to have an IC_50_ value of 0.530 (r^2^: 0.9655) on α-glycosidase, as reported by Kızıltaş et al. (2024).

## 4 Conclusions

*Rheum* species have great importance due to their rich contents of bioactive metabolites, however, according to the latest advances, several *Rheum* species are under immense risk and have been considered as “threatened” due to overharvesting and habitat destruction. Therefore, suggestions for good applications to cultivate them have been made. Besides, the discovery of novel *Rheum* sp. with wide biological activities is promising in many industries such as food, medicine, and cosmetics. According to the LC-MS/MS results, the abundant and major phenolic compounds detected in *R*. *telianum* were gallic acid, catechin, gentisic acid, tannic acid, epicatechin gallate, rutin, isoquercitrin, hesperidin quercitrin, astragalin, nicotiflorin, and acacetin. Here, it was evaluated that *R*. *telianum* extracts possess promising antioxidant activity, by having characteristics of the *Rheum* genus and Polygonaceae family. Industrially important metabolites of the species were revealed in remarkable quantity. This study can be concluded as a preliminary study and evaluated as seminal for future activity investigation of *R*. *telianum* species. The results clearly show that *R*.*telianum* plant has antioxidant effect, reducing ability and radical scavenging capacities. This plant has positive effects in the treatment of T2DM, AD, glaucoma, neurodegenerative and metabolic diseases due to its significant phenolic and flavonoid contents. In addition, this plant, which is widely used in folk medicine and ethnobotany, has the potential to be an important natural resource in the pharmaceutical and food industries. As a conclusion, the phytochemical content of this plant has been determined, antioxidant capacity has been revealed and which some its metabolic enzyme inhibition effects determined. We also hope that these great results, which obtained regarding this endemic plant will constitute a basic step and will be a guide for future studies. We also believe that this research will lead to many studies including molecular and detailed *in vivo* studies.

## Sample Availability

The authors do not provide samples of the substances.

## CRediT authorship contribution statement

**Ahmet Zafer Tel:** Resources, Methodology, Investigation. **Kubra Aslan:** Methodology, Investigation, Conceptualization. **Mustafa Abdullah Yılmaz:** Supervision, Formal analysis, Conceptualization. **İlhami Gulcin:** Writing – review & editing, Supervision, Resources, Project administration, Investigation, Conceptualization.

## Declaration of competing interest

The authors declare that they have no known competing financial interests or personal relationships that could have appeared to influence the work reported in this paper.

## Data Availability

The data that has been used is confidential.
